# Population-based colorectal cancer risk prediction using a SHAP-enhanced LightGBM model

**DOI:** 10.3389/fonc.2025.1575844

**Published:** 2025-07-17

**Authors:** Guinian Du, Hui Lv, Yishan Liang, Jingyue Zhang, Qiaoling Huang, Guiming Xie, Xian Wu, Hao Zeng, Lijuan Wu, Jianbo Ye, Wentan Xie, Xia Li, Yifan Sun

**Affiliations:** ^1^ Department of Laboratory Medicine, Eighth Affiliated Hospital of Guangxi Medical University, Guigang City People’s Hospital, Guigang, Guangxi, China; ^2^ Department of Laboratory Medicine, The People Hospital of Laibin, Laibin, Guangxi, China; ^3^ Department of Endocrinology, The People Hospital of Guigang, Guigang, Guangxi, China; ^4^ The Office of Administration, Liuzhou Municipal Liutie Central Hospital, Liuzhou, Guangxi, China

**Keywords:** Colorectal cancer, Risk prediction, Machine learning, LightGBM model, Early diagnosis

## Abstract

**Background:**

Colorectal cancer (CRC) is a highly frequent cancer worldwide, and early detection and risk stratification playing a critical role in reducing both incidence and mortality. we aimed to develop and validate a machine learning (ML) model using clinical data to improve CRC identification and prognostic evaluation.

**Methods:**

We analyzed multicenter datasets comprising 676 CRC patients and 410 controls from Guigang City People’s Hospital (2020-2024) for model training/internal validation, with 463 patients from Laibin City People’s Hospital for external validation. Seven ML algorithms were systematically compared, with Light Gradient Boosting Machine (LightGBM) ultimately selected as the optimal framework. Model performance was rigorously assessed through area under the receiver operating characteristic (AUROC) analysis, calibration curves, Brier scores, and decision curve analysis. SHAP (SHapley Additive exPlanations) methodology was employed for feature interpretation.

**Results:**

The LightGBM model demonstrated exceptional discrimination with AUROCs of 0.9931 (95% CI: 0.9883-0.998) in the training cohort and 0.9429 (95% CI: 0.9176-0.9682) in external validation. Calibration curves revealed strong prediction-actual outcome concordance (Brier score=0.139). SHAP analysis identified 13 key predictors, with age (mean SHAP value=0.216) and CA19-9 (mean SHAP value=0.198) as dominant contributors. Other significant variables included hematological parameters (WBC, RBC, HGB, PLT), biochemical markers (ALT, TP, ALB, UREA, uric acid), and gender. A clinically implementable web-based risk calculator was successfully developed for real-time probability estimation.

**Conclusions:**

Our LightGBM-based model achieves high predictive accuracy while maintaining clinical interpretability, effectively bridging the gap between complex ML systems and practical clinical decision-making. The identified biomarker panel provides biological insights into CRC pathogenesis. This tool shows significant potential for optimizing early diagnosis and personalized risk assessment in CRC management.

## Introduction

Colorectal cancer (CRC) is the third most common cancer worldwide, with the third highest morbidity and second highest mortality rate of all malignant tumors (1, 2). Despite its high mortality rate, early detection of CRC markedly enhances patient prognosis, with the five-year survival rate increasing from less than 15% in advanced stages to over 90% in early-stage disease. Consequently, the improvement of early screening rates for CRC is extremely urgent (3, 4). However, all the existing screening modalities have several disadvantages. Colonoscopy, though considered the gold standard for CRC screening, is highly invasive and has poor patient compliance, requiring considerable medical resources, which greatly limits its wide use (5, 6). Serum biomarkers, including carcinoembryonic antigen (CEA) and carbohydrate antigen 19-9(CA19-9), are convenient to test; however, their sensitivity and specificity are suboptimal, and positivity rates have been less than 30% in early-stage CRC, thus not being good enough for clinical screening (7, 8). In view of these limitations, there is an emerging trend to towards leveraging routine clinical data combined with machine learning(ML) for developing affordable and non-invasive CRC risk prediction models.

The core concentration toward deep learning (DL) and ML within artificial intelligence (AI) has very significantly enhanced the tumor prediction to catch the most minute clinical pattern. While the predictive accuracy and clinical applications of such AI-driven models vary significantly, notable advancements have been observed in recent years. For instance, recent studies have developed real-time DL models using white-light and image-enhanced endoscopy, demonstrating high accuracy in assessing tumor invasion depth (9).Moreover, AI algorithms have been used to predict lymph node and liver metastases, thus facilitating risk stratification and prognosis (10). Systematic reviews highlight the role of AI in screening, diagnosis, and treatment, thus supporting personalized care approaches in CRC (11). In addition, large cohort studies, such as the China Kadoorie Biobank, have provided valid risk models that incorporate demographic and lifestyle factors for CRC prediction. This development underlines the ever-growing participation of AI in CRC management by reinforcing the assessment of risk and treatment strategies (12, 13).

More novel approaches have also been developed, including CRC prediction models based on blood routine and biochemical indicators, providing a new pathway for early CRC screening with good sensitivity and accuracy (14). The integration of proteomic features with polygenic and non-genetic factors also greatly improved the accuracy of risk prediction and thus enabled personalized screening (15, 16). Finally, AI DL technology has also been applied in predicting the postoperative recurrence of CRC by analyzing the tumor microenvironment and immune cell densities, which provides a useful supplementary tool for clinical treatment decisions (17, 18). Despite these advancements, the landscape of CRC AI prediction models remains diverse, with considerable variations in predictive accuracy and clinical applicability. Many existing models are based on conventional statistical approaches and often lack rigorous external validation. Crucially, there is a distinct scarcity of research focusing on interpretable ML models in CRC prediction that can provide transparent insights into their decision-making processes. This interpretability is vital for clinical adoption, as it builds trust and enables clinicians to understand the rationale behind a prediction.

In this study, we address these gaps by developing a machine learning-based prediction model that systematically compares seven distinct algorithms to identify high-risk patients during follow-up care. We aimed to comprehensively evaluate algorithm performance and validate the most clinically reliable model. Unlike many previous studies, our approach emphasizes bridging the gap between complex computational analysis and practical bedside application. To achieve this, we developed an interactive web calculator that generates individualized risk profiles, enabling dynamic clinical decision-making at the point of care. This methodology enhances precision oncology by aligning prognostic tools with routine clinical practice, thereby holding significant potential for improving surveillance efficiency and long-term outcomes for CRC survivors.

## Methods

### Data source and study population

Research data was sourced from the Guigang City People’s Hospital, encompassing clinical information of patients who underwent treatment for CRC between 2020 and 2024. Rigorous de-identification procedures were implemented to safeguard the patients’ personal and sensitive information, thereby ensuring their privacy without the need for additional informed consent. Conducting the study in accordance with the principles outlined in the Declaration of Helsinki, approval was granted by the Ethics Committee of Guigang City People’s Hospital (approval number: E2023-001-23).

Adopting a retrospective design, this study adhered to the Transparent Reporting of a Multivariate Prediction Model for Individual Prognosis or Diagnosis (TRIPOD) statement to ensure transparency and reproducibility. For model construction, training (n = 651) and test (n = 435) cohorts were assembled from CRC patients recorded in the database. To validate the model externally, a separate cohort was selected from 463 patients treated at Laibin City People’s Hospital. Specifically, 168 CRC patients who received treatment between January 2023 and November 2024 and 295 control individuals were chosen for this validation cohort.

### Inclusion and exclusion criteria

Inclusion criteria were: 1) Confirmed CRC diagnosis per histopathology; 2) Availability of complete laboratory indices required for model development. Exclusion criteria included non-CRC comorbidities or recurrent hospital admissions (only data from the first admission were analyzed to prevent redundancy).

### Variable extraction

Data were systematically extracted from the electronic medical records of Guigang City People’s Hospital and Laibin City People’s Hospital. The variables included demographic information (age and sex), and a range of laboratory indices, including C-reactive protein, white blood cells (WBC), lymphocyte count (LC), neutrophil count, red blood cells (RBC), hemoglobin (HGB) levels, platelet count (PLT), alanine aminotransferase (ALT), aspartate aminotransferase, alkaline phosphatase, total protein (TP), albumin (ALB), globulin, creatinine (CRE), urea (UREA), and uric acid (UA), Additionally, tumor markers such as CEA and CA19–9 were included. The manual extraction process was employed to ensure the accuracy of the data.

### Sample size

In the realm of research, especially pertaining to clinical prediction models, there is no universally accepted standard for determining the appropriate sample size. Many models within this domain adopt the principle of 10 events per variable as a benchmark for assessing effective sample size. This principle stipulates a minimal ratio of 10 occurrences of the study event to the number of variables included. During our initial evaluation, we established that the number of features to be incorporated into our CRC risk prediction model would not surpass 13. Recognizing the importance of sufficient data for developing a reliable ML model, we deemed it essential to include at least 140 CRC patients. Our study leveraged a training cohort dataset comprising 651 CRC patients, which comfortably meets the threshold necessary for constructing a robust model.

### Quantification and statistical analysis

The data was processed and analyzed using R 4.4.1. Significance was set at p < 0.05. Missing data was handled using multiple imputation, excluding variables with >20% missing values. Normally distributed data was summarized with mean ± standard deviation and analyzed using the t-test. Non-normal data was summarized with median and interquartile range and analyzed using the Mann–Whitney U test. A heatmap showed variable correlations. Categorical variables were presented as frequencies/percentages and analyzed using the chi-square or Fisher’s test.

The data preprocessing pipeline was implemented using the recipes package in R, applying steps to remove variables with >20% missing values, eliminate zero-variance and near-zero-variance predictors, apply one-hot encoding to categorical variables, and address multicollinearity. The preprocessing workflow was developed on training data and applied consistently to test and validation datasets to prevent data leakage.

For feature selection, LASSO regression with cross-validation was implemented using the glmnet package. To determine the optimal regularization parameter, 10-fold cross-validation was employed, optimizing for area under the ROC curve. Lambda.1se (the value giving the most regularized model with error within one standard error of the minimum) was selected rather than lambda.min to prioritize model parsimony and reduce overfitting risk.

For hyperparameter tuning, random grid search with 5-fold cross-validation was used across seven machine learning algorithms: Decision Tree (DT), K-Nearest Neighbor (KNN), Light Gradient Boosting Machine (LightGBM), Random Forest (RF), Extreme Gradient Boosting (XGBoost), Support Vector Machine (SVM), and Multi-Layer Perceptron (MLP). For each model, 20 random hyperparameter combinations were generated within defined search spaces. The LightGBM model’s hyperparameters included tree depth (1-3), trees (100-500), and learning rate (10^-3^-10^-1^); XGBoost’s included tree depth (1-3), learning rate (10^-3^-10^-1^), and sample size (0.8-1.0); Random Forest’s included trees (200-500) and minimum node size (20-50); Decision Tree’s included tree depth (3-7) and cost complexity (10^-6^-10^-3^); KNN’s included neighbors (3-11); SVM’s included cost (10^-5^-10^5^) and sigma (10^-4^-10^-1^); and MLP’s included hidden units (15-24) and penalty (10^-3^-1). Configurations were selected using the one-standard-error rule to balance performance and complexity.

The models were evaluated on an external dataset using ROC curves, AUROC, calibration curves, and decision curve analysis. The best-performing model was interpreted using SHAP (SHapley Additive exPlanations). Finally, the LightGBM model was used to create a web-based CRC risk prediction calculator.

## Results

### Patient characteristics

Our baseline analysis encompassed a total of 676 CRC patients and 410 controls, randomly selected from the study population. **Table 1** presents their baseline characteristics. The median age across the entire cohort was 56.0 (range, 16.0–93.0) years. Gender distribution revealed that females constituted 29.7% (323 individuals), while males made up the majority at 70.3% (763 individuals). Notably, CRC patients tended to be older compared to the control group, with a median age of 63.5 years versus 50.0 years for controls. This age difference was statistically significant (p < 0.001), highlighting the increased prevalence of CRC in older populations.

**Table 1:** Comparison of baseline characteristics between Colorectal Cancer and Normal groups.

Clinical factors	Overall	Normal	Colorectal Cancer	P.value
	(N=1086)	(N=410)	(N=676)	
age (year)				<0.001
Median (Min, Max)	56.0 (16.0, 93.0)	50.0 (30.0, 59.0)	63.5 (16.0, 93.0)	
CEA				<0.001
Median (Min, Max)	3.19 (0.210, 33400)	2.10 (0.400, 14.6)	4.35 (0.210, 33400)	
CA199				<0.001
Median (Min, Max)	10.6 (0.600, 8740)	7.50 (0.600, 70.3)	12.7 (0.600, 8740)	
WBC				<0.001
Median (Min, Max)	6.70 (1.20, 29.7)	6.05 (2.60, 11.7)	7.41 (1.20, 29.7)	
LYMPH				<0.001
Median (Min, Max)	1.59 (0.140, 4.90)	2.07 (0.890, 4.90)	1.30 (0.140, 4.24)	
RBC				<0.001
Median (Min, Max)	4.63 (1.65, 7.53)	5.07 (3.14, 7.53)	4.22 (1.65, 6.97)	
HGB				<0.001
Median (Min, Max)	130 (31.0, 177)	145 (94.0, 177)	116 (31.0, 167)	
PLT				<0.001
Median (Min, Max)	266 (53.0, 1170)	255 (98.0, 640)	275 (53.0, 1170)	
ALT				<0.001
Median (Min, Max)	15.0 (1.00, 192)	21.0 (2.00, 128)	12.0 (1.00, 192)	
AST				<0.001
Median (Min, Max)	20.0 (8.00, 246)	22.0 (11.0, 99.0)	19.0 (8.00, 246)	
TP				<0.001
Median (Min, Max)	70.0 (36.7, 89.3)	74.7 (62.5, 89.0)	66.2 (36.7, 89.3)	
ALB				<0.001
Median (Min, Max)	42.1 (19.0, 52.2)	45.6 (38.0, 52.2)	38.7 (19.0, 51.9)	
GLB				<0.001
Median (Min, Max)	27.7 (13.7, 57.2)	29.2 (20.9, 41.3)	26.3 (13.7, 57.2)	
A/G				<0.001
Median (Min, Max)	1.50 (0.500, 2.59)	1.60 (1.10, 2.10)	1.40 (0.500, 2.59)	
UREA				<0.001
Median (Min, Max)	4.30 (0.940, 18.6)	4.53 (2.02, 17.8)	4.10 (0.940, 18.6)	
CRE				<0.001
Median (Min, Max)	79.5 (30.0, 791)	85.0 (46.0, 471)	73.0 (30.0, 791)	
UA				<0.001
Median (Min, Max)	336 (44.0, 840)	386 (124, 749)	293 (44.0, 840)	
gender				<0.001
female	323 (29.7%)	34 (8.3%)	289 (42.8%)	
male	763 (70.3%)	376 (91.7%)	387 (57.2%)	

In terms of biomarkers, CRC patients exhibited significantly altered levels compared to controls. WBC, LC, RBC, HGB, PLT, ALT, TP, ALB, UREA, CRE, and UA all showed notable differences between the two groups. For instance, the median WBC count was higher in CRC patients at 7.41 × 10^9^/L compared to 6.05 × 10^9^/L in controls (p < 0.001). Similarly, RBC count was lower in CRC patients (4.22 × 10^12^/L) than in controls (5.07 × 10^12^/L), with a p-value of <0.001. These biomarker discrepancies underscore the physiological impact of CRC on various blood components and organ functions.

**Figure 1:**
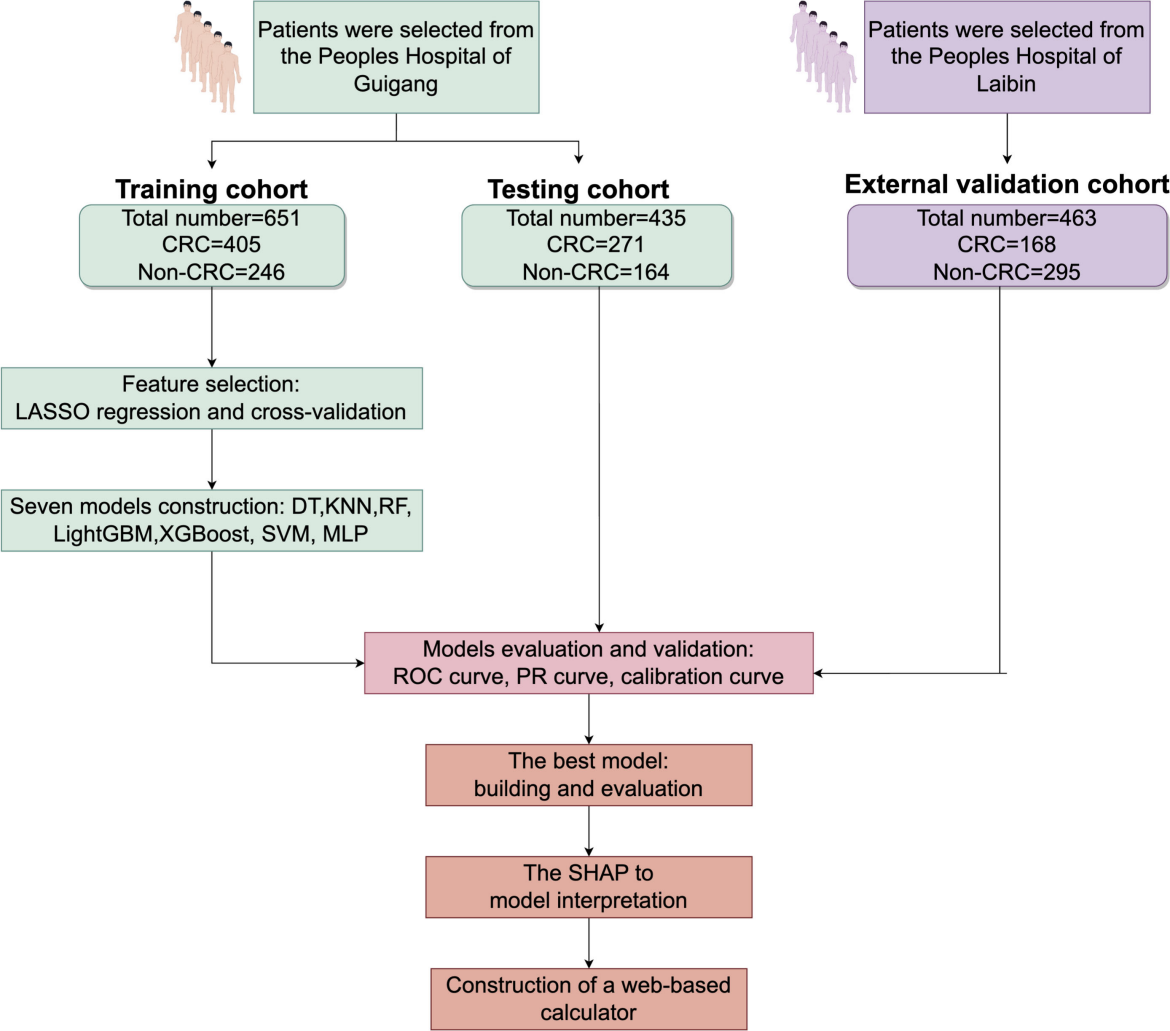
Research process for screening CRC patients CRC, colorectal cancer; LASSO-LR, Least Absolute Shrinkage and Selection Operator-Logistic Regression; DT, Decision Tree; KNN, K-Nearest Neighbor; LightGBM, Light Gradient Boosting Machine; RF, Random Forest; XGBoost, Extreme Gradient Boosting; SVM, Support Vector Machine; MLP, Multilayer Perceptron; SHAP, Shapley Additive Explanations.

Regarding tumor markers, CEA and CA19–9 levels were significantly elevated in CRC patients. The median CA19–9 level was 12.7 U/mL in CRC patients, compared to 7.50 U/mL in controls (p < 0.001). This elevation in CA19–9 is indicative of its potential as a diagnostic and prognostic biomarker for CRC, as higher levels often correlate with tumor presence and progression. **Figure 1** provides an overview of the screening and study process.

### Correlation between the study features

To elucidate the relationships among the study features, we generated a heatmap of the feature correlation matrix using the corrplot package in R. The outcomes are displayed in **Supplementary Figure S1**, and the analysis revealed several significant correlations. Notably, there was a negative correlation between age and TP levels, indicating that as age increases, TP levels tend to decrease. This finding suggests a potential age-related decline in protein synthesis or an increase in protein degradation, which could have implications for the overall health and nutritional status of older individuals. Other notable correlations were also identified, providing insights into the interplay of various factors in CRC risk and progression.

### Feature selection

**Figure 2:**
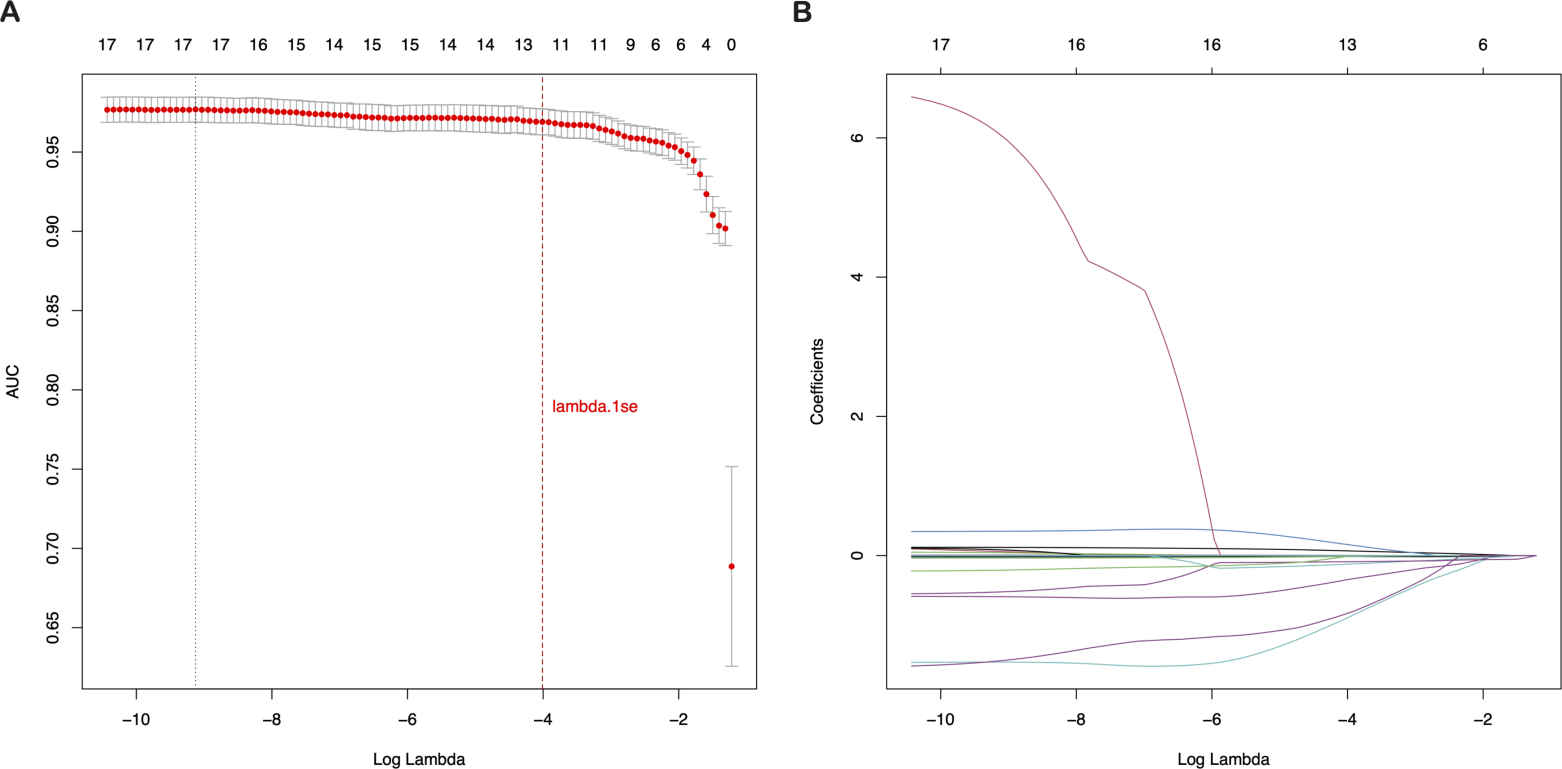
Selection of clinical features based on LASSO regression with cross-validation **(A)** LASSO coefficient profiles of the 19 texture features. **(B)** Tuning parameter (λ) selection using LASSO penalized LR with 10-fold cross-validation. LASSO, Least Absolute Shrinkage and Selection Operator; LR, Logistic Regression.

Employing the glmnet package in R, we conducted a LASSO regression cross-validation analysis to identify key predictors for CRC risk. With the dependent variable set as CRC risk prediction, we considered 19 features of the study population as independent variables. The results, illustrated in **Figure 2**, showed that at the lambda.1se tuning parameter value of γ = 0.018123, 13 distinct features were selected: age, CA19-9, WBC, LC, RBC, HGB, PLT, ALT, TP, ALB, UREA, UA, and gender. These features were incorporated into the prediction model, taking into account their clinical relevance and expert opinions. This meticulous feature selection process was crucial for refining the model to concentrate on the most influential predictors of CRC risk, thereby enhancing its predictive accuracy and clinical utility.

### Models’ construction and validation

**Figure 3:**
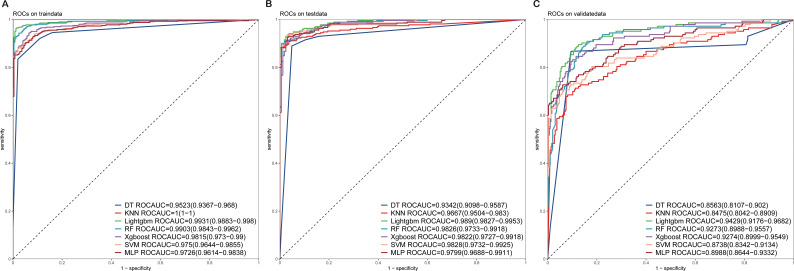
ROC curves of the test and validation sets of seven ML models **(A)** ROC curves of the train set. **(B)** ROC curves of the test set. **(C)** ROC curves of the validation sets. ROC, receiver operating characteristic; ML, machine learning.

**Figure 4:**
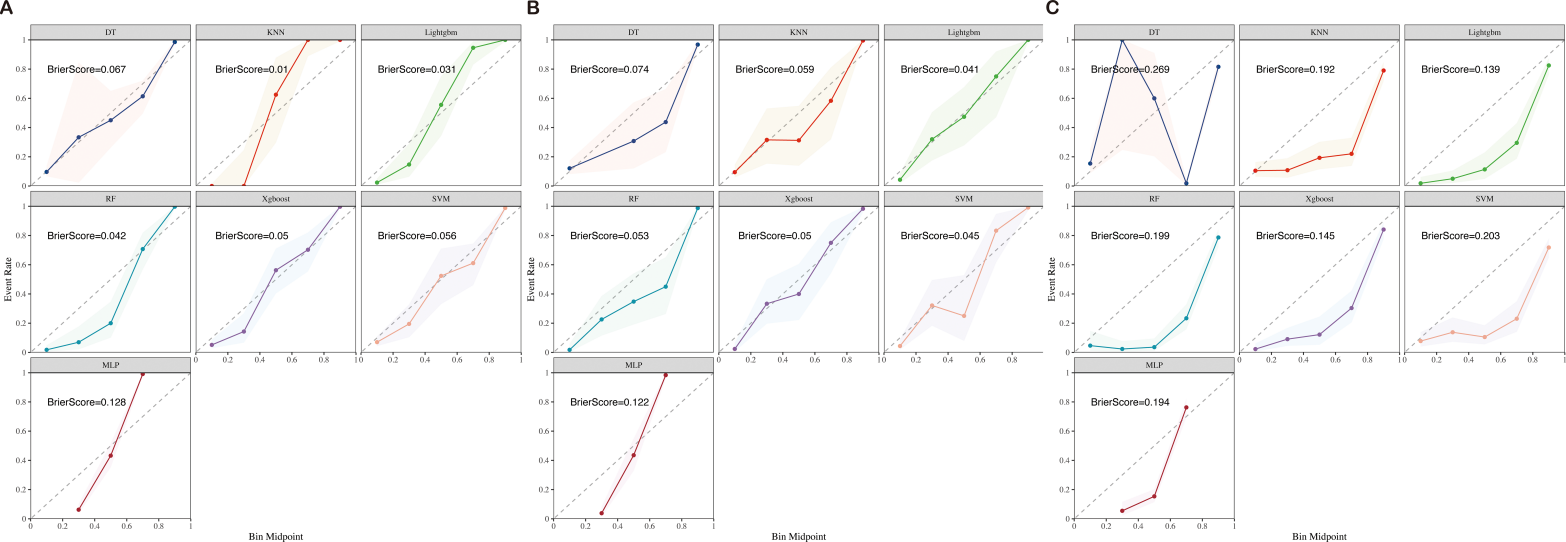
Training **(A)**, test **(B)**, and validation **(C)** calibration curves of seven ML models (DT, KNN, LightGBM, RF, XGBoost, SVM, and MLP) ML, machine learning; DT, Decision Tree; KNN, K-Nearest Neighbor; LightGBM, Light Gradient Boosting Machine; RF, Random Forest; XGBoost, Extreme Gradient Boosting; SVM, Support Vector Machine; MLP, Multilayer Perceptron.

The 13 filtered features were subsequently integrated into a variety of ML models, including LASSO-Logistic Regression (LASSO-LR), DT, KNN, LightGBM, RF, XGBoost, SVM, and MLP. To identify the optimal model, parameter tuning was conducted using 5-fold cross-validation, and multiple rounds of model training were executed. The optimal hyperparameter configurations for each model are provided in **Supplementary Table S1** in the **Supplementary Materials**. The detailed performance metrics for these seven ML models in both the training and external validation cohorts are presented in **Figure 3**, **Figure 4** and **Table 2**, respectively. In the training cohort, all models demonstrated an AUROC exceeding 0.75. Notably, the KNN model achieved the highest AUROC, reaching 1, whereas the DT model exhibited the lowest AUROC of 0.9523, presented in **Figures 3** and **Supplementary Figure S2**.

**Table 2:** Performance comparison of different ML models on the training, test, and validation sets across multiple evaluation metrics.

Model	Accuracy			Sensitivity			Specificity			F1 Score			ROC AUC		
	External Validation	Testing	Training	External Validation	Testing	Training	External Validation	Testing	Training	External Validation	Testing	Training	External Validation	Testing	Training
DT	0.893	0.913	0.889	0.867	0.889	0.835	0.905	0.951	0.980	0.841	0.927	0.904	0.856	0.934	0.952
KNN	0.724	0.926	1	0.811	0.908	1	0.681	0.957	1	0.657	0.939	1	0.848	0.967	1
Lightgbm	0.829	0.945	0.972	0.93	0.93	0.963	0.780	0.970	0.988	0.78	0.955	0.977	0.943	0.989	0.993
MLP	0.801	0.936	0.92	0.804	0.911	0.904	0.800	0.976	0.947	0.726	0.946	0.934	0.899	0.980	0.973
RF	0.854	0.926	0.955	0.909	0.915	0.943	0.827	0.945	0.976	0.802	0.939	0.963	0.927	0.983	0.99
SVM	0.815	0.926	0.917	0.762	0.897	0.879	0.841	0.976	0.980	0.729	0.938	0.930	0.874	0.983	0.975
Xgboost	0.792	0.929	0.940	0.909	0.93	0.943	0.736	0.927	0.935	0.741	0.942	0.951	0.927	0.982	0.981

ML, machine learning; KNN, K-Nearest Neighbor; RF, Random Forest; LightGBM, Light Gradient Boosting Machine; SVM, Support Vector Machine; XGBoost, Extreme Gradient Boosting; MLP, Multilayer Perceptron; DT, Decision Tree.

Upon assessing the external validation cohort, the LightGBM model displayed the highest AUROC of 0.9429. The remaining six ML models, ranked in descending order of AUROC, were as follows: XGBoost model (0.9274), RF model (0.9273), MLP model (0.8988), SVM model (0.8738), KNN model (0.8475), and DT model (0.8563). Pairwise statistical comparisons of AUROC values between different models using the DeLong test revealed that LightGBM significantly outperformed all other models (p < 0.05, **Supplementary Table S2**). The calibration curves for the various models, presented in **Figure 4**, all demonstrated satisfactory calibration. **Table 3** presents the Brier scores for all models across training, testing, and external validation cohorts. Specifically, the LightGBM model exhibited marginally superior calibration compared to the other seven models, with a Brier score of 0.139.

**Table 3:** The Brier scores for all models across training, testing, and external validation cohorts.

Model	Training cohort	Testing cohort	External validation cohort
xgboost	0.050	0.050	0.145
KNN	0.010	0.059	0.192
svm	0.056	0.045	0.203
lightgbm	0.031	0.041	0.139
Decision Tree	0.067	0.074	0.269
Random Forest	0.042	0.053	0.199
mlp	0.128	0.122	0.194

Further analysis using decision curve analysis revealed that all seven models, except for the DT and KNN models, which showed poor performance, possessed clinical utility. These findings suggest that the AI prediction models, particularly the LightGBM and XGBoost models, have the potential to be valuable tools in the prediction of CRC risk.

### Best model building and evaluation

In the external validation cohort for CRC, the LightGBM model emerged as the superior choice, demonstrating remarkable discrimination and calibration capabilities when benchmarked against other models. Specifically, it exhibited the highest AUROC of 0.9429 and a more favorable (lower) Brier score. To gain deeper insights into the model’s workings, we utilized the SHAP method, which allowed us to elucidate and visualize the contributions of various features to the model’s predictions. In addition, we developed an online web-based calculator to facilitate prospective predictions, enhancing the practical utility of our model in a clinical setting.

### Model interpretations

The SHAP method presents a holistic framework for interpreting the predictions made by our AI model, ensuring the provision of consistent and locally precise attribute values, termed SHAP values, for each feature integrated within the predictive model. Higher SHAP values signify an increased likelihood of CRC. The importance of predictor variables in predicting CRC can be comprehended as the cumulative effect of each variable’s attribution on the overall risk associated with the outcome.

**Figure 5:**
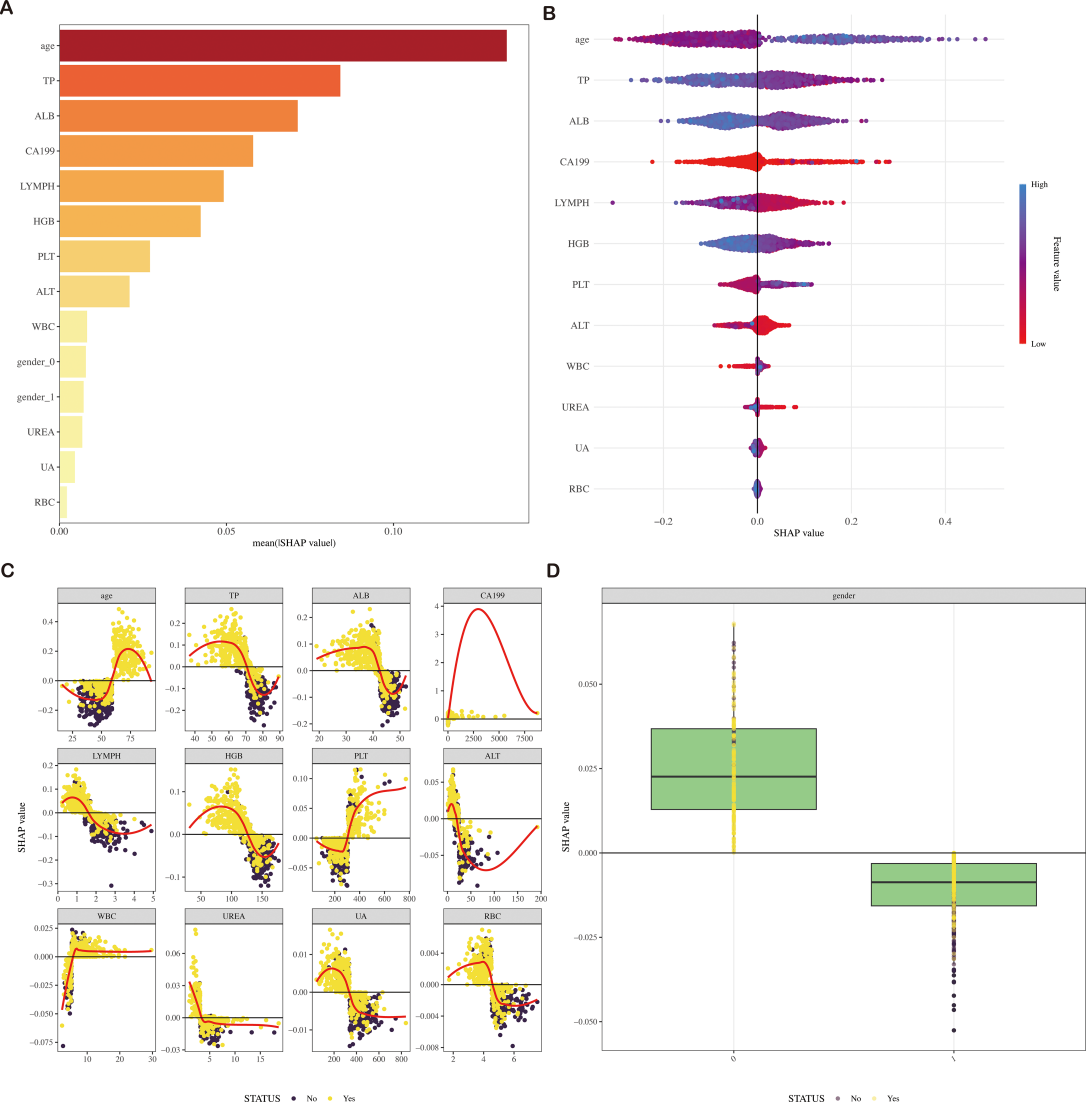
SHAP diagram of the LightGBM model **(A)** SHAP value ranking of the variables in the model. **(B)** SHAP honeycomb diagram of the LightGBM model. **(C)** SHAP value change trend diagram of continuous variables in the LightGBM model. **(D)** Box plot of SHAP values for categorical variables in the LightGBM model. SHAP, Shapley Additive Explanations; LightGBM, Light Gradient Boosting Machine.

In our study, 13 features were employed to construct the LightGBM model. **Figure 5** and **Supplementary Figure S3** visually rank these features based on their mean absolute SHAP values. The LightGBM model ranks the features according to their importance, including age, CA19-9, WBC, LC, RBC, HGB, PLT, ALT, TP, ALB, UREA, UA, and gender, indicating that an increase in these features’ values is associated with a higher risk of CRC.

### Evaluation of the model

In our assessment of the AI prediction model for CRC, the LightGBM algorithm exhibited robust discriminatory power within the training cohort, achieving an AUROC of 0.9931, with a 95% confidence interval (CI) ranging from 0.9883–0.998. Consistently, **Figure 3** showed that the model maintained its discriminatory ability in the external validation cohort, yielding an AUROC of 0.9429 (95% CI: 0.9176–0.9682).

### Application of the model

**Figure 6:**
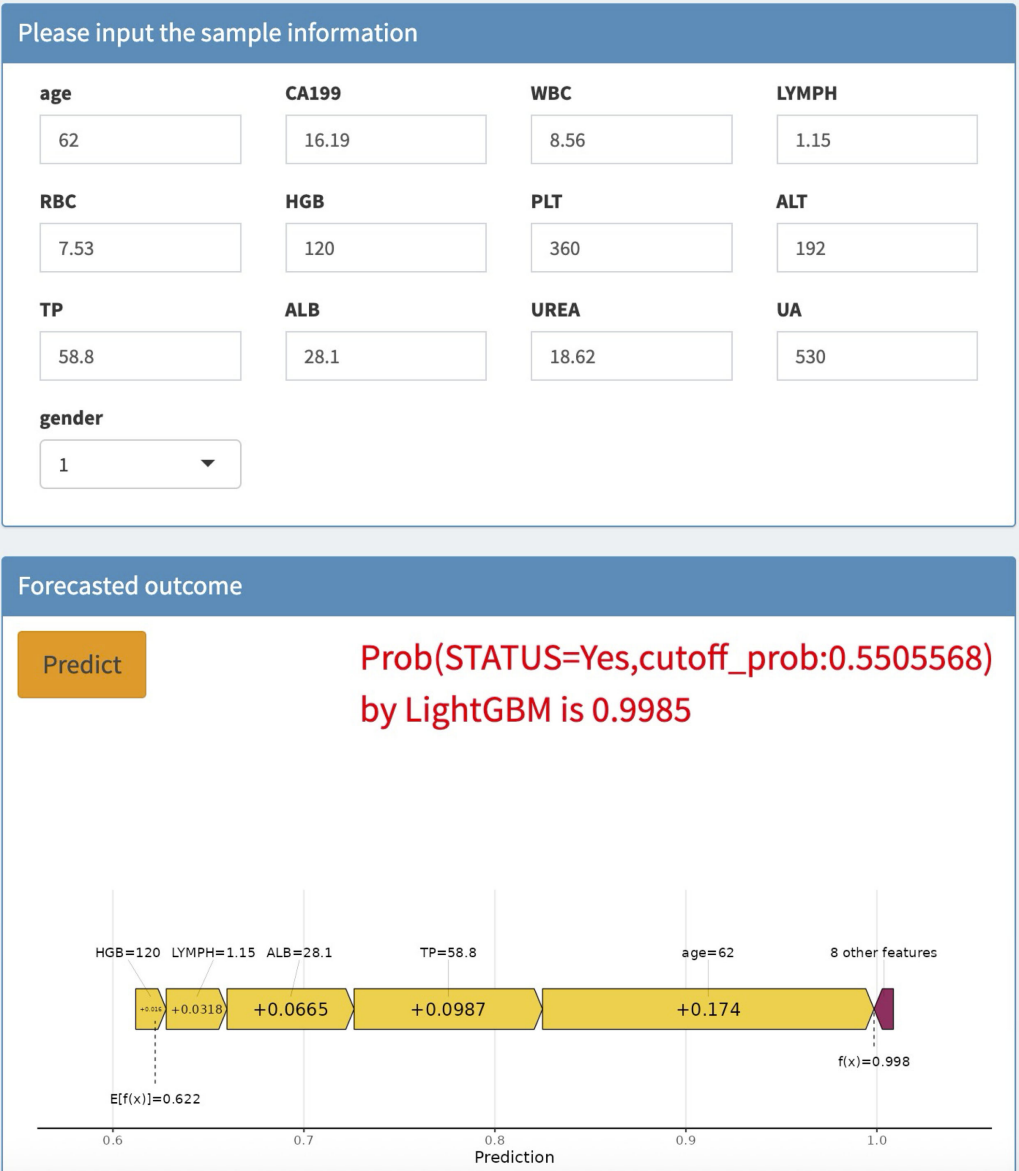
Case of website usage.

To enhance the convenience and effectiveness of the constructed model, we developed a web-based tool (accessible at: https://joezhicool.shinyapps.io/LightGBM/) that streamlines the process of utilizing the model for clinical decision-making. For example, consider a hypothetical 62-year-old individual with the following laboratory values: CA19-9 = 16.19 U/mL, TP = 58.8 g/L, ALB = 28.1 g/L, LC = 1.15 × 10^9/L, and HGB = 120 g/L. By inputting these values into the web-based calculator, the predicted probability of CRC for this individual is calculated as 0.9994, as shown in **Figure 6**. This example demonstrates the practical application of our model in assessing CRC risk based on readily available clinical data.

## Discussion

In this study, we explored the potential of ML algorithms in developing early warning models for CRC risk prediction using clinical data sourced from Guigang City People’s Hospital. Seven distinct ML algorithms were employed to establish predictive models aimed at identifying patients with a heightened likelihood of developing CRC. Following a rigorous series of training and validation procedures, the LightGBM model emerged as the most optimal choice for visualizing clinical risk predictions. This model demonstrated remarkable predictive capabilities in both the training and external validation cohorts, showcasing good discrimination, calibration, and clinical applicability. To facilitate the practical application of the selected model, we have also developed a web-based calculator that can assist doctors in making informed decisions during clinical consultations.

AI-based methods for CRC prediction have a number of advantages over traditional statistical methods. Although a number of studies tried to develop clinical risk prediction models for CRC, most of them were based on conventional statistical approaches and lack external validation (3, 19). There is a scarcity of research based on interpretable ML models in CRC prediction. Our study, therefore, focused on the identification of CRC-related factors and the development of a model that could provide substantial relationships of CRC risk with age, CA19-9, WBC, LC, RBC, HGB, PLT, ALT, TP, ALB, UREA, UA, and gender.

Among these variables, age has been consistently identified as an independent risk factor for CRC mortality (20). A comprehensive meta-analysis encompassing many studies demonstrated that older patients face a significantly increased risk of CRC-related death. Similarly, previous research has established that individuals aged 45–50 years are at particular risk for CRC (21, 22). Regarding LC, elevated levels have been linked to CRC progression and poor prognosis, as they may indicate systemic inflammation or an ongoing immune response to the tumor (23).

In addition to the aforementioned factors, CA19–9 and TP levels have also been implicated in CRC risk and prognosis. CA19-9, a tumor-associated antigen, has shown promise as a diagnostic and prognostic marker for CRC, although its specificity and sensitivity may vary depending on the stage and location of the tumor (24). Elevated CA19–9 levels have been associated with advanced CRC stages and poor survival outcomes (25, 26). Conversely, TP, a marker of nutritional status and protein synthesis, has been found to decrease in CRC patients, particularly those with advanced disease or cachexia. Low TP levels may reflect malnutrition and impaired immune function, both of which can adversely affect CRC prognosis (27, 28).

The LightGBM model developed in our study, using LASSO regression cross-validation, exhibited significant predictive power in determining the likelihood of CRC among patients. This model holds potential for clinical implementation, as it can aid healthcare providers in enhancing their understanding of patient prognosis and facilitate the tailoring of treatment and care plans for CRC patients (28–30). However, further validation of the model’s utility in larger, multicenter studies is necessary to confirm its robustness and generalizability.

Our study has several limitations. Firstly, the reliance on region-specific and single-institution datasets restricts generalizability, as findings may not fully translate to broader populations or diverse healthcare systems, particularly given insufficient external validation across varied demographic and clinical contexts. Secondly, data quality issues - including missing values, recording inconsistencies, and potential measurement errors - introduce uncertainties that could compromise model robustness and reproducibility. Thirdly, the variable selection framework focuses predominantly on conventional clinical parameters while underrepresenting emerging biomarkers and genetic predictors, potentially overlooking critical biological dimensions. Methodologically, the analysis faces inherent trade-offs between employing classical statistical approaches that struggle with high-dimensional data versus machine learning techniques whose “black box” nature limits clinical interpretability. These constraints collectively suggest that while the findings provide meaningful insights, caution should be exercised in extrapolating results beyond the study’s specific parameters.

## Conclusions

In conclusion, our study contributes to the evolving field of CRC risk prediction by leveraging clinical data and advanced ML techniques. The LightGBM model, coupled with the web-based calculator, represents a promising tool for early detection and risk stratification of CRC patients. Future research should focus on refining and validating this model in diverse patient populations to improve CRC outcomes.

## Data Availability

The original contributions presented in the study are included in the article/**Supplementary Material**. Further inquiries can be directed to the corresponding authors.
