# Supplementary figures and images for: Population-based colorectal cancer risk prediction using a SHAP-enhanced LightGBM model
Source: Front Oncol. 2025 Jul 17;15:1575844. doi: 10.3389/fonc.2025.1575844 (PMC12310463; doi:10.3389/fonc.2025.1575844)

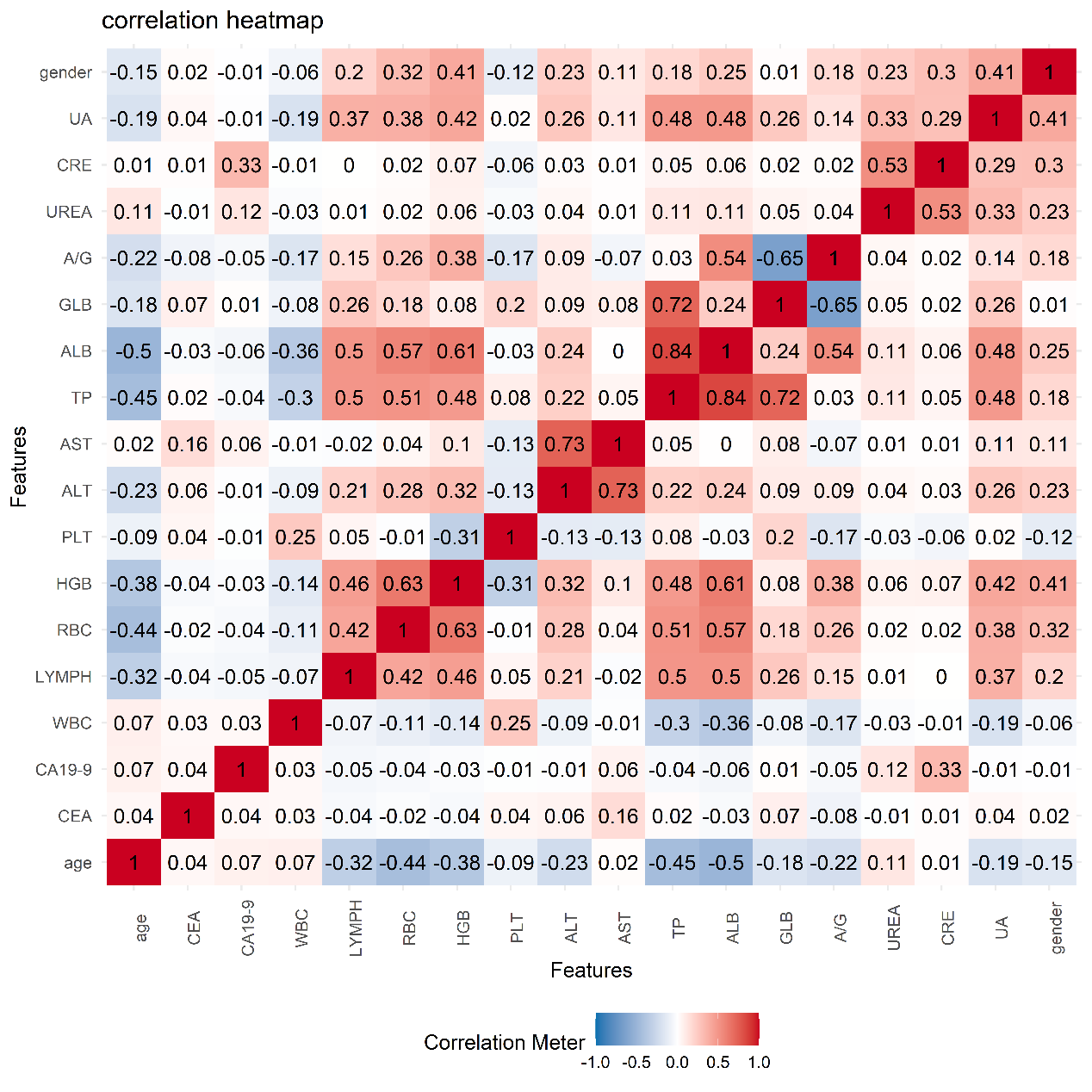

Supplement: Supplementary file 1 [file Image1.png]

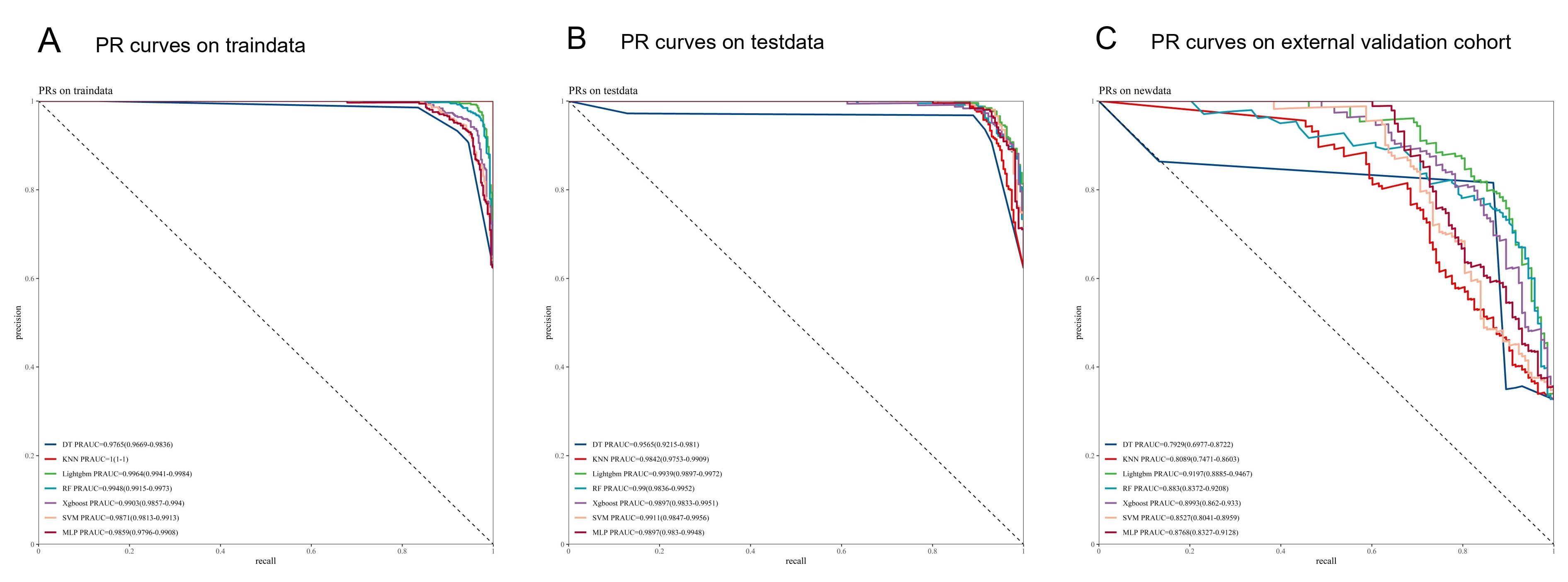

Supplement: Supplementary file 2 [file Image2.jpg]

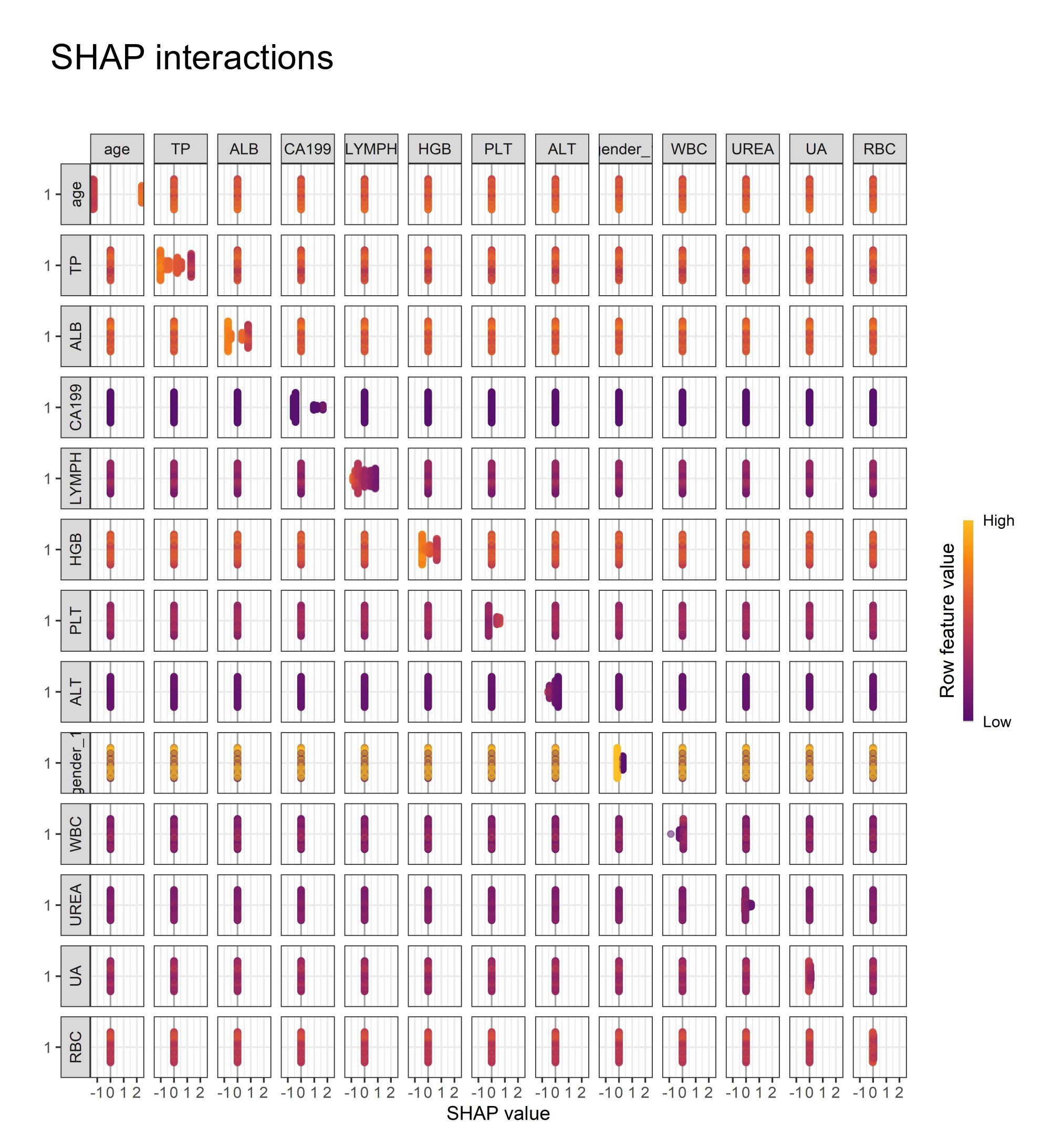

Supplement: Supplementary file 3 [file Image3.jpg]
